# Noise Robustness Evaluation of Time–Frequency Networks (TFNs) for Intelligent Mechanical Fault Diagnosis

**DOI:** 10.3390/s26144492

**Published:** 2026-07-15

**Authors:** Syed Khizar Zubair, Imran Shafi, Ahmet Caglar, Abdul Saboor Khan, Jamil Ahmad

**Affiliations:** 1 College of Electrical and Mechanical Engineering (CEME), National University of Sciences and Technology (NUST), Islamabad 46020, Pakistanimranshafi@ceme.nust.edu.pk (I.S.); 2 Department of Mechanical Engineering, Akdeniz University, 07070 Antalya, Turkey; 3 Department of Electrical Engineering and Information Technology, Otto-von-Guericke University, 39106 Magdeburg, Germany; 4 Department of Computer Science, Abdul Wali Khan University, Mardan 23200, Pakistan

**Keywords:** fault diagnosis, Time–Frequency Network, TFN, noise robustness, vibration analysis, deep learning, Gaussian noise, impulsive noise

## Abstract

Vibration-based mechanical fault diagnosis has become a critical research area, mostly driven by the need to improve equipment reliability and reduce unplanned downtime in industrial settings. Time–Frequency Networks (TFNs) have shown strong potential here, combining interpretable time–frequency transformations with deep learning classifiers in a single framework. This work reproduces the original TFN model from the recent literature and evaluates its noise robustness under additive Gaussian noise (10 dB, 0 dB, −5 dB SNR) and impulsive noise at the same levels, across five architectures: Backbone CNN, Random CNN, TFN-Chirplet, TFN-Morlet, and a squeeze-and-excitation attention CNN baseline. The evaluation protocol corrects two methodological issues identified during peer review of an earlier version of this work—window-level data leakage between train and test splits, and selection of the best-performing training epoch rather than a fixed final-epoch result—both of which are shown to materially affect reported outcomes. Under the corrected protocol, TFN-Morlet remains the most noise-robust architecture, with only a 19.09% accuracy drop from clean to −5 dB AWGN, approximately 15.5 percentage points better than Backbone CNN under the same conditions; an architectural anomaly reported in the earlier version of this study, in which mild noise appeared to improve an unconstrained CNN’s accuracy, was not reproduced under the corrected protocol and is shown to be an artifact of the original methodological issues. Per-class analysis and multi-model confusion matrices further reveal that misclassifications under severe noise are dominated by confusion between the same defect severity at different fault locations, rather than between different severities at the same location as previously reported. These results indicate that time–frequency-aware convolutional kernels improve both classification accuracy and noise resistance under rigorous, leakage-free evaluation, and that this robustness is not replicated by a generic attention mechanism alone.

## 1. Introduction

Mechanical systems such as motors, bearings, and rotating machinery are the heart of modern industrial operations, and when they fail the consequences range from costly downtime to genuine safety hazards [1,2,3]. This is why early fault detection has become a core concern in predictive maintenance and condition monitoring work [4].

Vibration signal analysis is probably the most widely used tool for this purpose [5]. Techniques such as Fourier Transform, Short-Time Fourier Transform (STFT), and Wavelet Transform have all been applied extensively to extract fault-related features from raw vibration data [6,7,8]. However, there is a significant practical limitation to these methods, as they heavily depend on expert knowledge and manual feature engineering, which makes them hard to scale or adapt to modern factory environments [9].

Deep learning, and CNNs in particular, have significantly altered this paradigm by enabling automatic feature extraction from the signal directly, without the need for hand-crafted pipelines [10,11,12]. It is worth noting that CNN architectures were not created for fault diagnosis in particular. The origins of these architectures trace back to diverse fields, drawing heavily from spatial pattern recognition in computer vision [13], temporal feature alignment in speech processing [14], and decision space exploration in deep reinforcement learning [15], before their adaptation to 1D mechanical signal analysis. While this progression to cross-domain applications significantly enhanced the diagnostic performance of rotating machinery, model interpretability remains a critical challenge. Standard CNNs still function as primarily opaque “black boxes,” leaving the industrial engineers unable to map learned representations back to the physical mechanical principles [16].

To resolve this limitation, Time–Frequency Networks (TFNs) integrate time–frequency transformations directly into the network architecture itself by bypassing external pre-processing pipelines [17]. Instead of relying on unconstrained, randomly initialized convolutional filters, TFNs leverage the parameterized time–frequency kernels. This design allows the network to isolate the physically meaningful spectral structures without sacrificing diagnostic performance baseline [18].

Our evaluation builds directly upon the TFN framework introduced by Chen et al. in “TFN: An interpretable neural network with time-frequency transform embedded for intelligent fault diagnosis” [17]. While original work established the mathematical foundations and demonstrated interpretability of TFNs under the idealized, laboratory-controlled conditions, resilience of the parameterized kernels against corrupted input signals remained unexamined. We first replicate the core TFN model according to the structural configurations and kernel formulations detailed in [17] and validate our implementation against their reported baseline benchmarks. Following the successful replication, we extend analysis by systematically introducing additive white Gaussian noise (AWGN) across a range of signal-to-noise ratios (SNRs). This stress-testing simulates the severe environmental disturbances—such as sensor cross-talk and mechanical vibrations—characteristic of operational industrial settings.

The main contributions of this work are summarized as follows:

- Successful reproduction of the original TFN framework proposed by Chen et al. [17].
- Validation on clean vibration signals of baseline performance.
- Systematic evaluation of the noise robustness across multiple SNR levels (10 dB, 0 dB, and −5 dB) under the both additive Gaussian and impulsive noise.
- Comparative analysis of five diagnostic models including a squeeze-and-excitation attention CNN baseline—under extreme signal corruption.
- Identification of the most noise-resistant TFN architecture for actual factory-floor deployment.
- Identification and correction of the test–train leakage and the evaluation-protocol issue in an earlier version of this study and transparent account of how corrected protocol changes reported results.

There are three specific research questions behind our investigation:

1. Do TFN-based models outperform the conventional CNN models under noisy conditions?
2. Which TFN kernel provides the highest robustness to Gaussian noise?
3. How does the model accuracy degrade as signal-to-noise ratio decreases?

The remainder of the study is organized systematically to present our framework. Section 2 contextualizes this work within the existing literature on traditional and deep-learning-based fault diagnosis. Section 3 details methodology, dataset parameters and noise injection frameworks by specifically introducing a corrected file-level data split and an evaluation protocol is designed to eliminate selection bias present in the earlier studies. Section 4 provides experimental outcomes and comparative analysis by focusing on how the revised protocol shifts previously reported architectural findings. Finally, Section 5 summarizes our conclusion and outlines potential avenues for future research.

## 2. Literature Review

### 2.1. Traditional Fault Diagnosis Methods

Historically, the backbone of vibration-based fault diagnosis has been a classical signal processing using FFT, STFT, wavelet transforms and related techniques [5,6,7,19]. Methods are well understood and can extract meaningful frequency-domain and transient information from raw signals, but they come with real practical cost. For example, someone with domain expertise has to design features manually, which can limit how far they can scale in automated or smart factory settings [1,8]. Table 1 summarizes the main trade-offs.

### 2.2. Deep Learning-Based Diagnosis

Manual feature engineering problems are largely solved by CNN-based approaches; ResNet and LSTM variants, for example, have pushed the classification accuracy considerably high by directly learning from the raw signal data [11,12,20,21], and this shift away from hand-crafted pipelines has been a major development in the field over the last decade. Standard CNNs are black boxes, as they offer very little insight in what the network actually learned or why the particular classification is made; to end users, it is a serious limitation when industrial engineers are trying to understand and trust the system [16,22]. For scenarios that involve sequential dependencies or fluctuating operating conditions, researchers have leveraged recurrent architectures, which have demonstrated resilience against noisy or mislabeled data [23].

### 2.3. Interpretable Neural Networks

Parallel research has attempted to address the interpretability problem without sacrificing performance, mostly by baking knowledge of signal processing directly into the network structure [24,25,26]. In general deep learning literature, interpretability methods span a wide range, including perturbation-based approaches [24]. The paradigm includes methods like contrastive explanation using pertinent negatives [27], routing path analysis through internal data [28] and dissection of formal networks to quantify hidden-layer concepts [29]. For visual interpretability, techniques such as saliency maps [30], Grad-CAM [31], axiomatic attribution [32] and prototype-based reasoning [33] have become prominent standards.

The main issue is that most of these models are designed on data for 2D images. When applied to 1D vibration transients, underlying assumptions break down, because fundamentally the signal structure is different. This pushed researchers towards domain-specific interpretable architectures, such as SincNet [34], Wavelet Kernel Networks [18] and W-CNN [35]. Broader frameworks have followed by including WhiteningNet [36], semi-supervised hybrid autoencoders [37] and resonance frequency band localization networks [38]. However, it is noted in both [39,40] that the majority of these models still rely on real-valued kernels by leaving them unable to fully capture complex time frequency energy distributions.

### 2.4. Modern Noise-Robust Architectures

In contrast, an active branch of the literature focuses on achieving noise robustness through architectural complexity instead of interpretability. For instance, lightweight 1D-CNNs trained with direct noise injection can achieve over 97% accuracy on the CWRU benchmark, even at a severe −10 dB SNR [41]. Attention-based architectures combining soft-thresholding, self-activation, and self-attention mechanisms have reported over 92% accuracy across a −9 dB to 9 dB noise range [42]. Multi-scale quaternion CNNs combined with bidirectional GRUs and cross self-attention feature fusion report near-ceiling accuracy on several public bearing datasets [43], and adaptive shapelet-based models combining gated parallel CNNs with bidirectional LSTMs have been evaluated directly against Gaussian, uniform, and impulsive noise on CWRU [44]. These architectures generally report higher raw accuracy under severe noise than the TFN-based and CNN-based models evaluated in this study, but typically do not retain TFN’s interpretability guarantees, since their parameters are not constrained to follow physically motivated time–frequency kernel profiles. This study’s scope is therefore deliberately narrower: rather than pursuing benchmark-leading accuracy, it asks whether physically constrained, interpretable kernels provide meaningful noise robustness without sacrificing interpretability, using unconstrained and attention-augmented CNN baselines as the relevant comparison points.

### 2.5. Research Gap

Despite all of this progress, one area has always received surprisingly little attention: how well do these interpretable networks actually hold up when input signals are noisy? Most evaluations, including Chen et al.’s original TFN paper, were run under clean and laboratory-controlled conditions [17]. Notable exceptions exist; for instance, multibranch and multiscale CNN architectures have been directly evaluated under strong noise and variable-load conditions [20], but such noise-aware testing remains the exception rather than norm for interpretable and physically constrained architectures specifically. While this is a reasonable starting point, real industrial environments are not clean; sensor noise, mechanical vibration cross-talk and electrical interference are routinely encountered. Whether structural constraints that make TFNs interpretable also provide inherent noise robustness remains an unresolved question, one which this study investigates directly.

## 3. Proposed Method

### 3.1. Overall Workflow

**Figure 1:**
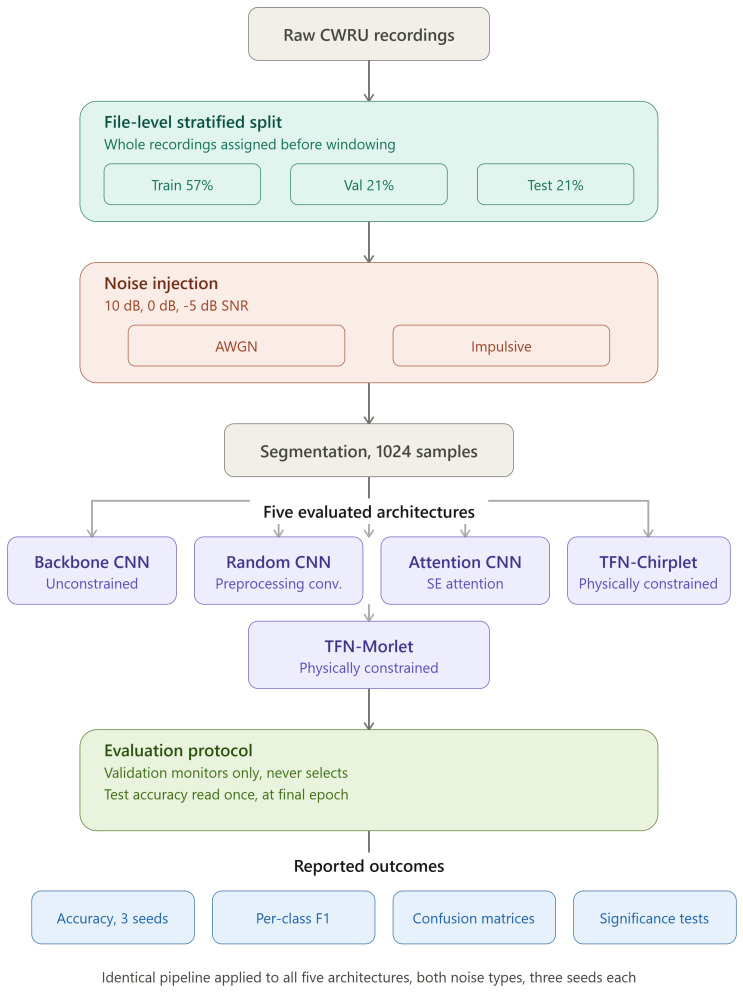
The proposed method flow diagram representing raw inputs, noise modeling (AWGN and impulsive), TFconv, segmentation and feature extraction, and the dense classification layers involved. The pipeline shown applies identically across all five evaluated architectures; the diagram’s annotations reflect the structure of the pipeline rather than the specific number of noise types or models compared, both of which were expanded in this revision (Section 3).

Figure 1 gives an overview of the full pipeline of our system. Raw vibration signals come in first, then noise is injected at the chosen SNR level, the signal gets segmented into fixed-length windows, and it then passes through the TFN layers before entering the classifier and evaluation stage.

### 3.2. Dataset Preparation and Domain Configurations

This study uses the Case Western Reserve University (CWRU) bearing dataset [21], which is one of the most widely used benchmarks in this field and is also the primary benchmark used by Chen et al. [17] for the original TFN framework. All noise robustness experiments in this study were conducted exclusively on CWRU. Earlier versions of this manuscript also reported clean-baseline results on a planetary gearbox dataset and an aerospace bearing dataset described in Chen et al. [17]; however, those datasets correspond to private experimental rigs used in the original authors’ laboratory and were never released publicly. As no independently verifiable source for that data exists, this revision restricts its scope to CWRU and reports the absence of public gearbox and aerospace benchmarks for this framework as a limitation (Section 5). This study uses the 12 kHz drive-end and fan-end CWRU recordings, rather than the 48 kHz recordings used by Chen et al. [17] for their main CWRU comparison; Chen et al. note that diagnostic accuracy on the 12 kHz signals is close to ceiling for most models, and switch to 48 kHz specifically to obtain a harder, more discriminating benchmark. This difference in difficulty is relevant when comparing absolute accuracy values against [17] in Section 4.

All signals were segmented into 1024-sample windows with no overlap. Unlike the segment-level splitting used in earlier work, the train, validation, and test sets in this study are constructed at the *file (recording) level*: entire raw recordings are assigned to exactly one of the three splits before any windowing takes place, and the assignment is stratified by fault class so each split contains examples of every class. This prevents windows drawn from the same continuous recording from appearing on both sides of the split, which would otherwise allow the model to partially recognize test-time signals it had effectively already seen during training. Files were split approximately 57% train/21% validation/21% test. Full dataset details are in Table 2.

### 3.3. Noise Injection Model

Noise was added to the clean vibration signals using a standard additive model:

(1) y(t)=x(t)+n(t)

where x(t) is the original signal and n(t) is the injected Gaussian noise sequence. We defined the SNR on the standard logarithmic scale:

(2) $$\mathrm{SNR}=10\log_{10}\;\left(\frac{P_{\mathrm{signal}}}{P_{\mathrm{noise}}}\right)$$

A total of three levels were tested that are 10 dB, 0 dB, and −5 dB. They roughly correspond to mild, moderate and severe industrial interference conditions.

In addition to AWGN, this study also evaluates the robustness against *impulsive noise*, which represents sparse, high-amplitude disturbances better. This also includes high-amplitude disturbances, such as electrical interference or the sudden mechanical shocks that are encountered on factory floors. The impulsive noise model combines a low-level background Gaussian floor with sparse high-amplitude spikes:

(3) $$n\left(t\right)=0.3\;\sigma_{b}\;g\left(t\right)+\sum_{k}a_{k}\;\delta \left(t-t_{k}\right)$$

where g(t) is the standard Gaussian noise, $\sigma_{b}$ represents the baseline noise standard deviation that is required in order to meet the target SNR, $t_{k}$ are randomly selected impulse locations (occurring with probability p=0.02 per sample) and $a_{k}∼N\left(0,{\left(8\sigma_{b}\right)}^{2}\right)$ are the impulse amplitudes. After adding the impulses, we rescale total noise power so that overall SNR still matches the target in Equation (2). This allows us to directly compare AWGN and impulsive noise conditions at each nominal SNR level.

For clarifying evaluation protocol, the noise is injected consistently into the training, validation and test signals for the given experimental condition instead of being added only at inference time as seen in Table 3. A separate model is completely trained from scratch for every combination of the architecture, noise type, SNR level and random seed instead of training one model on clean data and then evaluating it under multiple noise conditions. This means that each reported accuracy score in Table 4 and Table 5 belongs to an independently trained model, for which the entire training process, not just its evaluation, took place under that specific noise condition.

### 3.4. Model Architectures

All five models are trained and evaluated under similar conditions, so the performance difference can be attributed to architecture rather than the training setup.

- **Backbone CNN:** A standard CNN with randomly initialized convolution kernels and no structural constraint.
- **Random CNN:** A modified version of the above with an extra convolutional preprocessing layer inserted before the main feature extractor. Specifically, the Random CNN is the baseline architecture in Chen et al. [17], which uses an additional preprocessing convolution with non-parameterized kernels that serves as the direct structural contrast to the TFN variants.
- **Attention CNN:** A simplified squeeze-and-excitation (SE) attention baseline [45] matching the exact depth and channel structure of the Backbone CNN, with an SE block inserted after each convolutional stage. This adds only 6.2% additional parameters over Backbone CNN, so any accuracy difference is attributable to the attention mechanism rather than extra model capacity. This baseline is included to test whether a modern, learned attention mechanism alone—without TFN’s physically constrained kernels—can match TFN-level noise robustness.
- **TFN Chirplet:** This TFN variant uses parameterized kernels that are based on the Chirplet-transform [46,47].
- **TFN Morlet:** This TFN variant uses more complex Morlet wavelet kernels, following Chen et al.’s formulation [17,48].

In both TFN variants, the TF-conv layer runs a real–imaginary dual-kernel mechanism and computes a modulus feature map *h* as:

(4) $$h=\sqrt{h_{\mathrm{real}}^{2}+h_{\mathrm{imag}}^{2}}$$

This gives the network an explicit, interpretable spectral magnitude representation at each layer, rather than the opaque activations we get from standard convolutions [49].

### 3.5. Evaluation Metrics

We evaluate performance in this multi-class setup by using the generalized multi-class accuracy, calculated as:

(5) $$\mathrm{Accuracy}=\frac{\sum_{c=1}^{C}{\mathrm{TP}}_{c}}{\mathrm{Total}}$$

where ${\mathrm{TP}}_{c}$ represents the true positive count, which is calculated for each individual structural fault category *c* across total diagnostic class boundary *C*. Here, every prediction across all 10 fault classes in the CWRU dataset is counted. Accuracy drop score is used to quantify the robustness, the difference between clean and noisy performance at each SNR level:

(6) $$\mathrm{Drop}={\mathrm{Accuracy}}_{\mathrm{clean}}-{\mathrm{Accuracy}}_{\mathrm{noisy}}$$

A smaller drop represents more a noise-resistant model. That is basically the number that matters most for deployment decisions.

For assessing the class-specific behavior that an aggregate accuracy figure can hide, per-class precision, recall and F1-score were also computed for every model under every noise condition:

(7) $${\mathrm{Precision}}_{c}=\frac{{\mathrm{TP}}_{c}}{{\mathrm{TP}}_{c}+{\mathrm{FP}}_{c}},\;{\mathrm{Recall}}_{c}=\frac{{\mathrm{TP}}_{c}}{{\mathrm{TP}}_{c}+{\mathrm{FN}}_{c}},\;F1_{c}=\frac{2\cdot {\mathrm{Precision}}_{c}\cdot {\mathrm{Recall}}_{c}}{{\mathrm{Precision}}_{c}+{\mathrm{Recall}}_{c}}$$

The confusion matrices were also generated for all five models under the most severe noise condition to characterize the specific misclassification patterns that underlie each model’s aggregate accuracy.

For supporting the comparative claims that were made between models, a paired two-tailed *t*-test was performed across three random seeds and comparing the best-performing model against every alternative under each noise condition. Because of small sample size (*n* = 3), this test lacks the statistical weight to be definitive. Instead, we are presenting these results to simply back up the differences we observed in the averages.

### 3.6. Training Configuration

All five networks were trained under identical settings throughout the whole experiment. We used the Adam optimizer with a weight decay rate of $1\times 10^{-4}$, a fixed learning rate of 0.0001 [50], gradient norm clipping at a maximum norm of 1.0 to prevent occasional gradient explosions under high-noise conditions, and a batch size of 64 over 30 epochs, which is enough for all configurations to reach stable convergence. All noise robustness experiments were repeated and tested across three random seeds (42, 100, and 999) to assess result stability; accuracy and standard deviation are reported in Table 4.

All models were implemented in PyTorch and trained on a single GPU provided through a free Google Colab runtime. Backbone CNN, Random CNN, and Attention CNN each use four convolutional stages with 16, 32, 64, and 128 channels, respectively, a kernel size of 15 for the first convolutional layer and 3 for subsequent layers, followed by a fully connected classifier with hidden layer sizes of 256 and 64 before the final 10-class output layer. Attention CNN additionally inserts a squeeze-and-excitation block after each convolutional stage, using a channel-reduction ratio of 4. TFN-Chirplet and TFN-Morlet replace the first convolutional stage with a time-frequency convolutional layer of kernel size 11 and 32 intermediate channels, following the formulation in Chen et al. [17], with kernel parameters clamped to a physically valid range during training. All other architectural and training hyperparameters are identical across the five models to isolate the effect of architecture from the effect of training configuration.

Within each run, the validation set was used only to monitor training progress across epochs; it was never used to select which epoch’s weights to report. Accuracy reported for every model, condition and seed in this study is test-set accuracy measured once, after the final (30th) training epoch, instead of the highest accuracy that was observed across all epochs. This distinction matters because cherry-picking the single best epoch score is effectively testing the model over and over until you get the highest number. This artificially inflates the final accuracy and masks how the model actually generalizes to new data.

## 4. Results and Discussion

### 4.1. Baseline Validation Results

Before running the noise experiments, the reproduced models were checked against the clean-condition benchmark results from Chen et al. [17]. As described in Section 3, the file-level train/validation/test split means clean accuracy here is reported as the 3-seed mean (±standard deviation), consistent with the noise-condition reporting in Table 4, rather than as a single-run value. Table 3 shows the resulting metrics for all five models.

The TFN variants retain a clear accuracy advantage over all three non-TFN architectures even under clean conditions, reproducing the same architectural ranking reported by Chen et al. [17]. Absolute accuracy is substantially lower here than in [17] for every architecture; we attribute this gap primarily to the file-level train/test split adopted in this study, which removes the window-level leakage present in segment-level splits, such as the one used in the original TFN evaluation. A leakage-free split is expected to yield lower, but more trustworthy, accuracy, since the model can no longer partially recognize test-time windows drawn from recordings it was also trained on. The wider standard deviations compared to earlier single-run reporting reflect genuine sensitivity to which specific files are held out for testing under the corrected file-level split, and are discussed further in Section 4.

### 4.2. Noise Robustness Evaluation

Table 4 shows how each of the five models held up as AWGN SNR dropped from clean conditions down to −5 dB. Table 5 reports the equivalent results under impulsive noise (Section 3), included to test whether the observed ranking generalizes beyond Gaussian interference alone.

**Figure 2:**
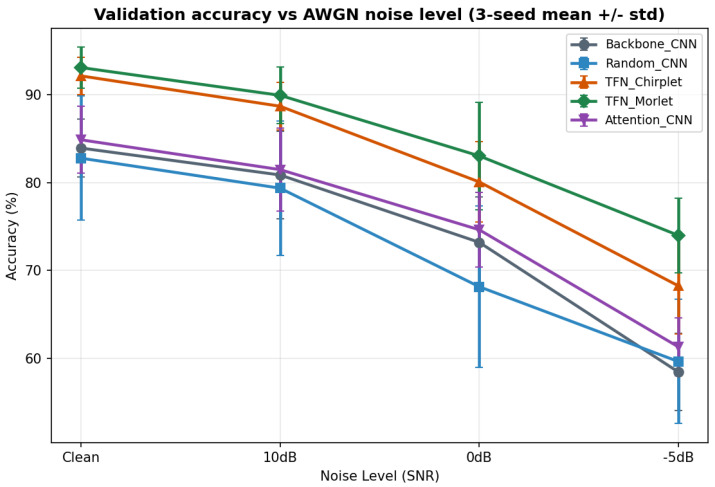
Validation accuracy vs. AWGN noise level across all five evaluated models. The standard deviation across the 3 random seeds is shown as error bars. Wider variance compared to earlier reporting reflects the corrected file-level train/test split.

This general trend is visible in Figure 2. TFN-Morlet and TFN-Chirplet degrade more gracefully than the three non-TFN architectures as noise worsens under AWGN. By −5 dB, the performance gap between TFN-Morlet and Backbone CNN has widened to approximately 15.5 percentage points.

### 4.3. Accuracy Drop Analysis

**Figure 3:**
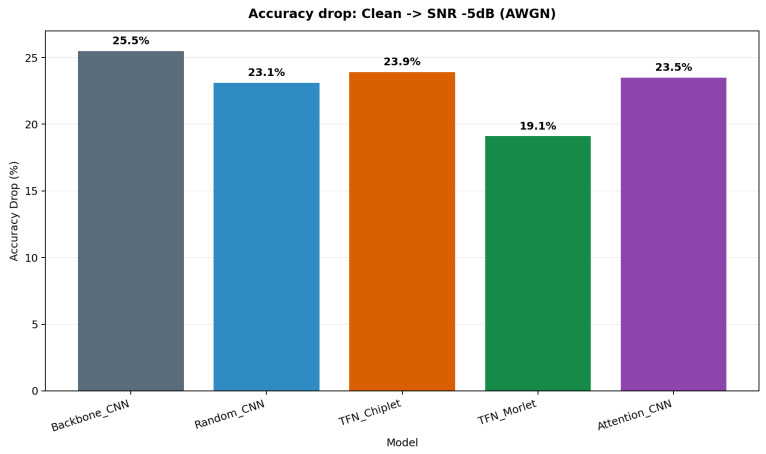
Accuracy drop comparison highlights the total degradation from clean to the −5 dB conditions under AWGN, across all five evaluated models.

In order to obtain a single number characterizing each model’s worst-case noise sensitivity, Table 6 records mean accuracy drop from the clean conditions to −5 dB under AWGN, visualized in Figure 3.

TFN-Morlet’s 19.09% drop is the smallest among the five models, approximately 4.8 points lower than TFN-Chirplet and 6.4 points lower than the Backbone CNN at the same noise level.

### 4.4. Discussion

#### 4.4.1. The Overall Trend and Corrected 10 dB Result

The main result confirms that the TFN-based models outperform conventional CNNs under the noise and that this advantage grows significantly as the SNR decreases. TFN-Morlet is the most robust of the the five architectures tested, retaining 73.98% accuracy at −5 dB AWGN (Table 4), consistent with expectation that physically constrained wavelet kernels focus only on frequency bands carrying fault information while suppressing out-of-band noise [17].

An earlier version of this study reported an anomaly at 10 dB, in which Backbone CNN’s accuracy appeared to increase under mild noise relative to clean conditions, attributed to noise acting as an implicit regularizer. Re-running the full experiment under the corrected file-level split and final-epoch reporting protocol (Section 3) shows that this effect does not hold: at 10 dB, every one of the five architectures—including both TFN variants—shows a small, ordinary accuracy decrease relative to clean conditions (approximately 3 percentage points on average; Table 4), with no architecture showing the asymmetric improvement previously reported. This indicates the originally observed anomaly was very likely an artifact of two compounding methodological issues in the earlier evaluation protocol: window-level data leakage between train and test splits, and reporting the best validation-epoch accuracy across training rather than the accuracy at a fixed, final evaluation point. Once both issues are corrected, noise behaves as expected at every SNR level tested, for every architecture.

#### 4.4.2. Robustness Under Impulsive Noise

Table 5 shows that the overall ranking observed under AWGN—TFN variants outperforming non-TFN architectures, with the gap widening under more severe noise—also holds under impulsive noise. One notable difference is that TFN-Chirplet matches or marginally exceeds TFN-Morlet under impulsive noise at 0 dB and −5 dB (85.03% vs. 84.59% and 79.68% vs. 79.66%, respectively), though paired testing (Table 7) shows this difference is not statistically distinguishable from chance. This is consistent with the Chirplet kernel’s localized time-frequency structure being well suited to sparse, spike-like interference, though we do not treat this as a confirmed advantage given the small effect size and seed count.

Random CNN shows markedly higher instability under impulsive noise than any other architecture, with a standard deviation of 11.01% at 0 dB and the lowest mean accuracy of any model at −5 dB (44.02%, a 35.66 point gap behind TFN-Chirplet, p=0.0085). This suggests that Random CNN’s unconstrained preprocessing convolution provides no particular benefit under sparse, high-amplitude interference, and may in fact be a source of instability relative to both the fully unconstrained Backbone CNN and the physically constrained TFN variants.

#### 4.4.3. Statistical Significance

Table 7 summarizes paired *t*-tests comparing the best-performing model against each alternative under every noise condition. TFN-Morlet’s advantage over Backbone CNN and Attention CNN is statistically significant (p<0.05) under every AWGN condition tested, including the clean baseline. The advantage over TFN-Chirplet is directionally consistent but does not reach significance under most of the conditions, consistent with both variants, which draw similar benefit from physically constrained kernels.

#### 4.4.4. Per-Class Behavior

Overall accuracy can easily mask the major weaknesses in specific categories. Table 8 reports the F1-scores per class at −5 dB AWGN for all five models. Two patterns stand out. First, every model is able to perform nearly perfect on the Normal class (F1 > 0.92) and comparatively poor on the Ball 0.014 (F1 between 0.148 and 0.418 across all five models). This indicates that this specific fault signature remains difficult to isolate under severe noise even for the most robust architecture tested. Second, TFN-Morlet’s advantage over Backbone CNN is not spread evenly across all ten classes. Instead, the gains are heavily concentrated in Outer race fault classes, where the F1 score jumps from 0.478–0.577 up to 0.804–0.876.

#### 4.4.5. Confusion Matrix Analysis

**Figure 4:**
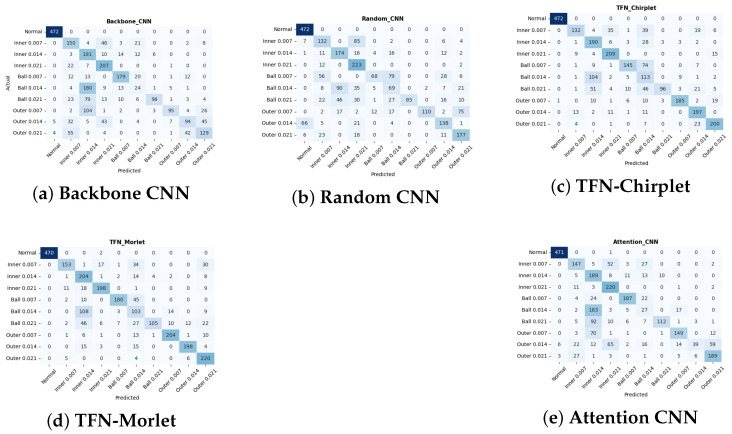
Confusion matrices for all five evaluated architectures at extreme −5 dB AWGN. The dominant misclassification pattern (Ball 0.014 is confused with Inner 0.014) is consistent across every model.

Figure 4 shows the confusion matrices for all five models at −5 dB AWGN, inspecting all jointly, rather than for a single model in isolation. This reveals a consistent misclassification pattern that was present in an earlier single-model observation. A previous version of this study also reported that misclassifications under severe noise reflected confusion between adjacent severities of the same fault location (e.g., a 0.014-inch inner race fault confused with a 0.021-inch inner race fault). Examining corrected confusion matrices across for all five architectures shows a different and more consistent dominant pattern: the Ball 0.014 fault is systematically misclassified as Inner 0.014 in every model tested, even in some cases catastrophically (e.g., 183 of 210 Ball 0.014 samples misclassified as Inner 0.014 under Attention CNN). This indicates that under severe noise, the dominant confusion is between *the same defect severity occurring at different fault locations*, instead of being between different severities at the same location. This suggests the that defect magnitude, instead of the defect position, becomes the dominant learned feature as signal quality degrades, a more specific characterization of the model failure than the original single-matrix analysis could support.

#### 4.4.6. Attention Without Physical Constraints

Despite matching the Backbone CNN’s exact depth and channel structure with only 6.2% parameter overhead from its SE attention blocks, the Attention CNN is unable to approach TFN-level robustness under any noise condition (Table 4 and Table 5). The accuracy and drop profile remains close to the Backbone CNN throughout and its disadvantage relative to the TFN-Morlet is statistically prominent in most conditions tested. This supports the understanding that TFN’s robustness stems specifically from the physically motivated kernel constraints, rather than any of the general benefit conferred by learned attention mechanisms alone.

## 5. Conclusions and Future Work

This work set out to achieve two main objectives. The first is to reproduce the TFN framework from Chen et al. [17], and the second is to push it into territory, which the original paper did not cover, specifically how these models actually behave when the input signals are noisy, as in a factory environment. Both objectives were met. This reproduction confirms the same architectural ranking that was reported by Chen et al. on CWRU, though the absolute clean-condition accuracy is lower across all five models under our corrected, file-level, leakage-free split. Section 4 argues this gap is itself evidence that leakage present in segment-level splits actually inflates reported accuracy. The noise robustness evaluation successfully produced clear and interpretable results.

The headline of the finding is straightforward. TFN-based models hold up exceptionally better than conventional CNNs as the noise increases and among all the five architectures tested, TFN-Morlet was standout, preserving the smallest accuracy drop and strongest performance at every noise tier from 10 dB to −5 dB under both the AWGN and impulsive noise. The primary reason, we argue, comes down to physics-constrained kernel structure. Morlet-based filters behave much like the adaptive bandpass filters and naturally suppress out-of-band noise, which CNNs that have unconstrained kernels simply cannot replicate and which also a generic attention mechanism without such constraints (Attention CNN) does not replicate. For engineers considering deployment in noisy industrial setups where SNR regularly drops to 0 dB or below, TFN-Morlet shows the strongest results among the architectures evaluated here. This recommendation is based on AWGN and impulsive noise injected into a single public benchmark dataset, and should be weighed alongside untested factors relevant to real deployment, including colored (non-white) noise profiles, non-stationary factory interference, variable operating loads and speeds, sensor-specific characteristics, and inference constraints on embedded or edge hardware.

There is also a broader takeaway. Interpretability and robustness are often treated as separate design goals, but the TFN results strongly suggest that the two can reinforce each other. Physically meaningful kernel constraints can buy us noise resistance more or less for free, so both goals are on the same page.

This study also illustrates a more general methodological point. An earlier iteration of this work reported an architectural anomaly under mild noise that did not survive correction once test–train leakage and an evaluation protocol vulnerable to selection bias were both fixed. Reporting this correction, rather than omitting it, is intended to demonstrate that the core claims of this study—TFN’s noise robustness advantage over conventional CNNs—hold under substantially more rigorous experimental conditions than those used in the original evaluation, including a corrected data split, an unbiased evaluation protocol, a second noise type, statistical significance testing, and a modern attention-based baseline.

Like any published study, this one has its limitations, and several directions seem worth pursuing from here:

- Extending noise robustness evaluation to additional public bearing and gearbox datasets to test whether the observed advantage of physically constrained kernels generalizes across mechanical domains, since the planetary gearbox and aerospace bearing data used in the original TFN study are not publicly available (Section 3).
- We benchmarked our model against the recent noise-robust architectures from broader fault diagnosis literature [41,42,43,44]. Although several models report higher raw accuracy on the CWRU dataset under severe noise, they typically lack the interpretability guarantees which TFN provides.
- Investigating transfer learning and domain adaptation across different mechanical datasets and machine types.
- Exploring deployment on micro-controller edge hardware, where real-time inference constraints impose a different set of trade-offs.
- Developing adaptive, self-tuning TFN architectures that can adjust their kernel parameters in response to changing noise environments.

## Figures and Tables

**Table 1:** Comparison of traditional fault diagnosis methods.

Method	Advantages	Limitations
FFT	Simple, fast	No time localization
STFT	Time-frequency analysis	Fixed resolution
Wavelet Transform	Multi-resolution	Parameter sensitive
EMD	Adaptive decomposition	Mode mixing

**Table 2:** Structural overview of the CWRU bearing dataset used in this study.

Dataset	Fault Classes	Samples/Class	Split (Train/Val/Test)
CWRU Bearing Dataset	10 classes	∼600–1200	57%/21%/21%

**Table 3:** Baseline clean accuracy on the CWRU bearing dataset (3-seed mean ± std).

Model	Clean Accuracy
Backbone CNN	83.91% ± 3.32%
Random CNN	82.76% ± 7.05%
TFN-Chirplet	92.12% ± 2.12%
TFN-Morlet	93.07% ± 2.35%
Attention CNN	84.85% ± 3.82%

**Table 4:** The validation accuracy under AWGN at different noise levels (3-seed mean ± std).

Model	Clean	10 dB	0 dB	−5 dB
Backbone CNN	83.91% ± 3.32%	80.85% ± 4.99%	73.19% ± 5.19%	58.44% ± 4.39%
Random CNN	82.76% ± 7.05%	79.35% ± 7.63%	68.14% ± 9.20%	59.63% ± 7.07%
TFN-Chirplet	92.12% ± 2.12%	88.67% ± 2.71%	80.06% ± 4.58%	68.24% ± 5.47%
TFN-Morlet	93.07% ± 2.35%	89.91% ± 3.19%	83.02% ± 6.11%	73.98% ± 4.25%
Attention CNN	84.85% ± 3.82%	81.46% ± 4.74%	74.62% ± 4.26%	61.30% ± 3.32%

**Table 5:** Validation accuracy under the impulsive noise at different noise levels, i.e., 3-seed mean ± std.

Model	10 dB	0 dB	−5 dB
Backbone CNN	80.39% ± 4.92%	74.07% ± 5.95%	61.78% ± 4.32%
Random CNN	78.37% ± 4.56%	66.99% ± 11.01%	44.02% ± 4.83%
TFN-Chirplet	90.36% ± 1.91%	85.03% ± 4.73%	79.68% ± 8.15%
TFN-Morlet	91.77% ± 1.30%	84.59% ± 3.01%	79.66% ± 5.95%
Attention CNN	79.90% ± 4.99%	74.03% ± 8.05%	58.22% ± 3.39%

**Table 6:** Mean accuracy drop from clean to −5 dB under AWGN.

Model	Mean Drop (%)
Backbone CNN	25.47
Random CNN	23.13
TFN-Chirplet	23.88
TFN-Morlet	19.09
Attention CNN	23.55

**Table 7:** Paired significance tests (best model vs. each alternative) at representative noise conditions. Asterisk denotes p<0.05.

Condition	Comparison	Mean Diff. (%)	*p*-Value	Sig.
Clean	TFN-Morlet vs. Backbone CNN	+9.16	0.0497	*
Clean	TFN-Morlet vs. Attention CNN	+8.22	0.0323	*
−5 dB AWGN	TFN-Morlet vs. Backbone CNN	+15.53	0.0065	*
−5 dB AWGN	TFN-Morlet vs. Random CNN	+14.34	0.0189	*
−5 dB AWGN	TFN-Morlet vs. TFN-Chirplet	+5.74	0.0605	
−5 dB Impulsive	TFN-Chirplet vs. Random CNN	+35.66	0.0085	*
−5 dB Impulsive	TFN-Chirplet vs. TFN-Morlet	+0.02	0.9935	

**Table 8:** F1-score per class at −5 dB AWGN (seed 42) for all five models.

Class	Backbone CNN	Random CNN	TFN-Chirplet	TFN-Morlet	Attention CNN
Normal	0.991	0.922	0.999	0.998	0.990
Inner 0.007	0.557	0.521	0.663	0.745	0.636
Inner 0.014	0.466	0.618	0.623	0.633	0.464
Inner 0.021	0.724	0.669	0.825	0.852	0.733
Ball 0.007	0.782	0.416	0.711	0.837	0.827
Ball 0.014	0.148	0.306	0.400	0.418	0.154
Ball 0.021	0.568	0.528	0.566	0.605	0.622
Outer 0.007	0.546	0.630	0.847	0.874	0.703
Outer 0.014	0.478	0.607	0.777	0.876	0.276
Outer 0.021	0.577	0.664	0.830	0.804	0.756
Macro avg	0.584	0.588	0.724	0.764	0.616

## Data Availability

The original data presented in the study are openly available in the Case Western Reserve University (CWRU) Bearing Data Center at https://engineering.case.edu/bearingdatacenter.

## References

[1] Li J., Wang X., Wu H. (2021). Rolling bearing fault detection based on improved piecewise unsaturated bistable stochastic resonance method. IEEE Trans. Instrum. Meas..

[2] Tao F., Qi Q., Liu A., Kusiak A. (2018). Data-driven smart manufacturing. J. Manuf. Syst..

[3] Chen H., Jiang B., Ding S.X., Huang B. (2022). Data-driven fault diagnosis for traction systems in high-speed trains: A survey, challenges, and perspectives. IEEE Trans. Intell. Transp. Syst..

[4] Lei Y., Yang B., Jiang X., Jia F., Li N., Nandi A.K. (2020). Applications of machine learning to machine fault diagnosis: A review and roadmap. Mech. Syst. Signal Process..

[5] Li R., He D. (2012). Rotational machine health monitoring and fault detection using EMD-based acoustic emission feature quantification. IEEE Trans. Instrum. Meas..

[6] Li Y., Xu M., Wei Y., Huang W. (2015). An improvement EMD method based on the optimized rational Hermite interpolation approach and its application to gear fault diagnosis. Measurement.

[7] Peng Z., Chu F. (2004). Application of the wavelet transform in machine condition monitoring and fault diagnostics: A review with bibliography. Mech. Syst. Signal Process..

[8] Li Y., Xu M., Liang X., Huang W. (2017). Application of bandwidth EMD and adaptive multiscale morphology analysis for incipient fault diagnosis of rolling bearings. IEEE Trans. Ind. Electron..

[9] Si Y., Wang Y., Zhou D. (2021). Key-performance-indicator-related process monitoring based on improved kernel partial least squares. IEEE Trans. Ind. Electron..

[10] Shao H., Jiang H., Zhang H., Liang T. (2018). Electric locomotive bearing fault diagnosis using a novel convolutional deep belief network. IEEE Trans. Ind. Electron..

[11] Wang Y., Liu R., Lin D., Chen D., Li P., Hu Q., Chen C.L.P. (2021). Coarse-to-fine: Progressive knowledge transfer-based multitask convolutional neural network for intelligent large-scale fault diagnosis. IEEE Trans. Neural Netw. Learn. Syst..

[12] Zhao X., Yao J., Deng W., Ding P., Ding Y., Jia M., Liu Z. (2022). Intelligent fault diagnosis of gearbox under variable working conditions with adaptive intraclass and interclass convolutional neural network. IEEE Trans. Neural Netw. Learn. Syst..

[13] Brunetti A., Buongiorno D., Trotta G.F., Bevilacqua V. (2018). Computer vision and deep learning techniques for pedestrian detection and tracking: A survey. Neurocomputing.

[14] Fayek H.M., Lech M., Cavedon L. (2017). Evaluating deep learning architectures for speech emotion recognition. Neural Netw..

[15] Silver D., Schrittwieser J., Simonyan K., Antonoglou I., Huang A., Guez A., Hubert T., Baker L., Lai M., Bolton A. (2017). Mastering the game of Go without human knowledge. Nature.

[16] Zhang Q.-S., Zhu S.-C. (2018). Visual interpretability for deep learning: A survey. Front. Inf. Technol. Electron. Eng..

[17] Chen Q., Dong X., Tu G., Wang D., Cheng C., Zhao B., Peng Z. (2024). TFN: An interpretable neural network with time-frequency transform embedded for intelligent fault diagnosis. Mech. Syst. Signal Process..

[18] Li T., Zhao Z., Sun C., Cheng L., Chen X., Yan R., Gao R.X. (2022). WaveletKernelNet: An interpretable deep neural network for industrial intelligent diagnosis. IEEE Trans. Syst. Man Cybern. Syst..

[19] Oppenheim A.V., Willsky A.S., Nawab S.H., Hernández G.M. (1997). Signals & Systems.

[20] Peng D., Wang H., Liu Z., Zhang W., Zuo M.J., Chen J. (2020). Multibranch and multiscale CNN for fault diagnosis of wheelset bearings under strong noise and variable load condition. IEEE Trans. Ind. Informat..

[21] Zhao Z., Li T., Wu J., Sun C., Wang S., Yan R., Chen X. (2020). Deep learning algorithms for rotating machinery intelligent diagnosis: An open source benchmark study. ISA Trans..

[22] Xi P.-P., Zhao Y.-P., Wang P.-X., Li Z.-Q., Pan Y.-T., Song F.-Q. (2019). Least squares support vector machine for class imbalance learning and their applications to fault detection of aircraft engine. Aerosp. Sci. Technol..

[23] Nie X., Xie G. (2021). A novel normalized recurrent neural network for fault diagnosis with noisy labels. J. Intell. Manuf..

[24] Ivanovs M., Kadikis R., Ozols K. (2021). Perturbation-based methods for explaining deep neural networks: A survey. Pattern Recognit. Lett..

[25] Fan F.-L., Xiong J., Li M., Wang G. (2021). On interpretability of artificial neural networks: A survey. IEEE Trans. Radiat. Plasma Med. Sci..

[26] Zhang Y., Tino P., Leonardis A., Tang K. (2021). A survey on neural network interpretability. IEEE Trans. Emerg. Top. Comput. Intell..

[27] Dhurandhar A., Chen P.-Y., Luss R., Tu C.-C., Ting P., Shanmugam K., Das P. (2018). Explanations based on the missing: Towards contrastive explanations with pertinent negatives. Proceedings of Advances in Neural Information Processing Systems.

[28] Wang Y., Su H., Zhang B., Hu X. Interpret neural networks by identifying critical data routing paths. Proceedings of the IEEE Conference on Computer Vision and Pattern Recognition.

[29] Bau D., Zhou B., Khosla A., Oliva A., Torralba A. Network dissection: Quantifying interpretability of deep visual representations. Proceedings of the IEEE Conference on Computer Vision and Pattern Recognition.

[30] Simonyan K., Vedaldi A., Zisserman A. (2014). Deep inside convolutional networks: Visualising image classification models and saliency maps. arXiv.

[31] Selvaraju R.R., Cogswell M., Das A., Vedantam R., Parikh D., Batra D. Grad-CAM: Visual explanations from deep networks via gradient-based localization. Proceedings of the IEEE International Conference on Computer Vision.

[32] Sundararajan M., Taly A., Yan Q. Axiomatic attribution for deep networks. Proceedings of the 34th International Conference on Machine Learning.

[33] Li O., Liu H., Chen C., Rudin C. (2018). Deep learning for case-based reasoning through prototypes: A neural network that explains its predictions. Proceedings of the AAAI Conference on Artificial Intelligence.

[34] Ravanelli M., Bengio Y. (2019). Interpretable convolutional filters with SincNet. arXiv.

[35] Ganguly B., Chaudhury S., Biswas S., Dey D., Munshi S., Chatterjee B., Dalai S., Chakravorti S. (2020). Wavelet kernel based convolutional neural network for localization of partial discharge sources within a power apparatus. IEEE Trans. Ind. Informat..

[36] Li J., Wang Y., Zi Y., Zhang Z. (2021). Whitening-Net: A generalized network to diagnose the faults among different machines and conditions. IEEE Trans. Neural Netw. Learn. Syst..

[37] Wu X., Zhang Y., Cheng C., Peng Z. (2021). A hybrid classification autoencoder for semi-supervised fault diagnosis in rotating machinery. Mech. Syst. Signal Process..

[38] Wang D., Chen Y., Shen C., Zhong J., Peng Z., Li C. (2022). Fully interpretable neural network for locating resonance frequency bands for machine condition monitoring. Mech. Syst. Signal Process..

[39] Zhao B., Cheng C., Tu G., Peng Z., He Q., Meng G. (2021). An interpretable denoising layer for neural networks based on reproducing kernel Hilbert space and its application in machine fault diagnosis. Chin. J. Mech. Eng..

[40] Michau G., Frusque G., Fink O. (2022). Fully learnable deep wavelet transform for unsupervised monitoring of high-frequency time series. Proc. Natl. Acad. Sci. USA.

[41] Hakim M., Omran A.A.B., Inayat-Hussain J.I., Ahmed A.N., Abdellatef H., Abdellatif A., Gheni H.M. (2022). Bearing fault diagnosis using lightweight and robust one-dimensional convolution neural network in the frequency domain. Sensors.

[42] Zhang Y., Lin L., Wang J., Zhang W., Gao S., Zhang Z. (2025). Attention activation network for bearing fault diagnosis under various noise environments. Sci. Rep..

[43] Liu H., Zhang F., Tan Y., Huang L., Li Y., Huang G., Luo S., Zeng A. (2024). Multi-scale quaternion CNN and BiGRU with cross self-attention feature fusion for fault diagnosis of bearing. arXiv.

[44] Hu W., Zhou H., Yang J. (2026). Interpretable and noise-robust bearing fault diagnosis for CNC machine tools via adaptive shapelet-based deep learning model. Machines.

[45] Hu J., Shen L., Sun G. Squeeze-and-excitation networks. Proceedings of the IEEE Conference on Computer Vision and Pattern Recognition.

[46] Yang Y., Zhang W., Peng Z., Meng G. (2013). Multicomponent signal analysis based on polynomial chirplet transform. IEEE Trans. Ind. Electron..

[47] Tu G., Dong X., Chen S., Zhao B., Hu L., Peng Z. (2020). Iterative nonlinear chirp mode decomposition: A Hilbert-Huang transform-like method in capturing intra-wave modulations of nonlinear responses. J. Sound Vib..

[48] Cohen M.X. (2019). A better way to define and describe Morlet wavelets for time-frequency analysis. NeuroImage.

[49] Andrearczyk V., Whelan P.F. (2016). Using filter banks in convolutional neural networks for texture classification. Pattern Recognit. Lett..

[50] Kingma D.P., Ba J. Adam: A method for stochastic optimization. Proceedings of the International Conference on Learning Representations (ICLR).

